# Evaluation of Round Window Stimulation Performance in Otosclerosis Using Finite Element Modeling

**DOI:** 10.1155/2016/3603207

**Published:** 2016-02-29

**Authors:** Shanguo Yang, Dan Xu, Xiaole Liu

**Affiliations:** ^1^School of Mechatronic Engineering, China University of Mining and Technology, Xuzhou 221116, China; ^2^Jiangsu Key Laboratory of Mine Mechanical and Electrical Equipment, China University of Mining and Technology, Xuzhou 221116, China

## Abstract

Round window (RW) stimulation is a new type of middle ear implant's application for treating patients with middle ear disease, such as otosclerosis. However, clinical outcomes show a substantial degree of variability. One source of variability is the variation in the material properties of the ear components caused by the disease. To investigate the influence of the otosclerosis on the performance of the RW stimulation, a human ear finite element model including middle ear and cochlea was established based on a set of microcomputerized tomography section images of a human temporal bone. Three characteristic changes of the otosclerosis in the auditory system were simulated in the FE model: stapedial annular ligament stiffness enlargement, stapedial abnormal bone growth, and partial fixation of the malleus. The FE model was verified by comparing the model-predicted results with published experimental measurements. The equivalent sound pressure (ESP) of RW stimulation was calculated via comparing the differential intracochlear pressure produced by the RW stimulation and the normal eardrum sound stimulation. The results show that the increase of stapedial annular ligament and partial fixation of the malleus decreases RW stimulation's ESP prominently at lower frequencies. In contrast, the stapedial abnormal bone growth deteriorates RW stimulation's ESP severely at higher frequencies.

## 1. Introduction

Middle ear implants (MEI) serving as an alternative treatment option for hearing loss have been a dynamic area of research during the last decade [1–5]. Unlike conventional hearing aids which use amplified sound pressure to compensate hearing impairment, the MEI always takes advantage of direct mechanical stimulation utilizing an implanted actuator to compensate hearing loss. For this reason, MEI can overcome the shortcomings of traditional hearing aids, such as higher sound distortion, limited amplification, noise and ringing, and discomfort [1].

The MEI commonly stimulates the ossicular chain; then the vibration is transmitted into the cochlea. In this process, the vibrational energy is transmitted into the cochlea through the oval window (OW). This form of MEI's application is called forward drive. However, coupling the transducer to an ossicle is difficult in ears with the middle ear disease, such as otosclerosis. To approach this issue, an alternative way of coupling sound to the cochlea by driving the round window (RW), called RW stimulation or reverse drive, was developed, and clinical studies on this novel MEI's application have been reported [6–9].

Nonetheless, clinical reports show a high variability regarding the degree of hearing compensation performance [10]. Besides, Beltrame et al. found that the actual functional gain provided by the RW stimulation is less than the theoretic expectancy [11]. To discern the factors that may cause the variability in efficacy of the RW stimulation, a lot of studies have been conducted in human temporal bone experiments. Experiments included variations such as different orientation, intervening materials between the RW and the actuator, overlaying materials, and pretension force applied on the actuator. In addition to the experimental measurements on temporal bones, the finite element models of the human ear were also used by Zhang and Gan [12] and Tian et al. [13] to investigate the effect of the actuator size and the coupling conditions, respectively. However, all of the variables are device-loading variables. To the authors' knowledge, there is still no report on the effect of patient-specific variables (hearing loss etiology, hearing loss configuration, etc.) on the RW stimulation performance.

Otosclerosis is a disease with abnormal growth of the ossicular bones in the middle ear. The typical changes of otosclerosis in the human ear are increased stiffness of the stapedial annular ligament [14–16], stapedial abnormal bone growth [17–19], and partial fixation of the malleus [16, 20]. These changes of ear components' properties also affect the hearing compensation performance of RW stimulation. Therefore, to figure out the influence of the otosclerosis on the RW stimulation is crucial for this type of MEI, especially for ascertaining the driving force of its actuator.

As noted above, although the device-loading variables that are leading to the variability in efficacy of the RW stimulation have been studied extensively, none of the effect concerning the otosclerosis has been reported. In this paper, we establish a 3D finite element (FE) model of the human ear to aid the analysis. By comparing the differential pressure across the partition of the cochlea under reverse and forward stimulation, the effect of otosclerosis related patient-specific variables on the performance of RW stimulation was investigated. The theoretical result is intended to support the optimization design of the actuator in order to insure sufficient compensation.

## 2. Materials and Methods

### 2.1. Finite Element Model of the Human Ear

A human middle ear geometric model was constructed based on a high resolution CT imaging data set of a human temporal bone (male, age 45, right ear). The model consists of the tympanic membrane (TM), ossicles (malleus, incus, and stapes), tendons, and ligaments. The corresponding FE mesh model was built using the FE preprocessing software Hypermesh (Altair Engineering, Tory, MI). The tympanic membrane and tympanic annulus were meshed by a total of 1569 three-node shell elements. The ossicles and suspensory ligaments/tendons were meshed by 14949 four-node tetrahedral solid elements.

The cochlea was modeled as a straight chamber with rigid wall filled with fluid. The chamber is divided by the basilar membrane (BM) and the osseous spiral lamina into two equal ducts: the scala vestibuli (SV) and the scala tympani (ST). The height of the fluid-filled SV and ST ducts was assumed to change linearly in height from 2.0 mm at the base to 0.4 mm at the apex, and the width changed linearly from 3.2 mm at the base to 0.65 mm at the apex. The length of both the SV and ST was assumed to be 37 mm, and the fluid volumes of the SV and ST were 92.315 mm^3^ and 93.270 mm^3^, respectively. These two fluid ducts were connected at the apical end of the fluid chamber via a hole representing the helicotrema. The helicotrema was modeled as a rectangular fluid passageway with dimensions of 0.65 × 1.6 mm. The model of the round window membrane (RWM) has a round shape with an area of 2.1 mm^2^, which is similar to the mean area of 2.08 mm^2^ reported by Atturo et al. [21]. To avoid complications of the analysis, Reissner's membrane and the micromechanical structure of the organ of Corti were ignored in this model. This simplification method of the cochlea has been commonly accepted for the study of cochlear mechanics [22, 23]. Besides, the width of the BM changed linearly from 150 *μ*m at the base to 500 *μ*m at the apex, and the thickness of the BM also changed linearly from 7.5 *μ*m at the base to 2.5 *μ*m at the apex [22]. At last, the BM was meshed by 482 four-node shell elements. The scala vestibuli and scala tympani were meshed by 17577 and 13802 eight-node hexahedral solid elements, respectively.

**Figure 1:**
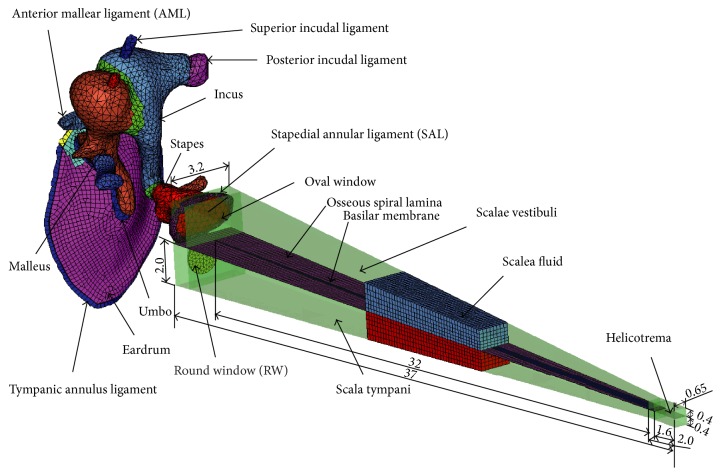
Finite element model of human ear with middle ear components and simplified cochlea.

The final established human ear FE model is shown in Figure 1. The characteristic dimensions of our human ear FE model's components are also listed in Table 1, which shows that the most dimensions of our model are within the range of published human ear data.

### 2.2. Material Properties

The materials of the middle ear system were assumed to be linear elastic. The material properties used for the FE model were listed in Table 2. These properties of the middle ear components were initially selected from published data [24–28]. Then, these values were turned by matching the simulated responses to the experimental responses. Poisson's ratio was assumed to be 0.3 for all of the components of the middle ear system [27]. The damping coefficients *α* and *β* for all materials of the middle ear system were taken as *α* = 0 s^−1^, *β* = 0.0001 s [27].

The material properties defined for the cochlea were elastic modulus, density, and Poisson's ratio. Similarly, the material properties of the cochlear components were initially assumed according to published literatures [13, 22]. Then, these parameters were checked and some of them modified in the validation process. The elastic modulus of the BM was 40 MPa at the base, 15 MPa at the middle, and 3 MPa at the apex. The elastic modulus of the RW membrane and osseous spiral lamina were set to 0.3 MPa and 1.41 × 10^4^ MPa, respectively. The density and Poisson's ratio of the osseous spiral lamina, RW, and BM were set to 1200 kg/m^3^ and 0.3, and the damping parameter *β* was assumed to be 1 × 10^−4^ and 5 × 10^−3^ for the BM and other solid components in the cochlea. The bulk modulus and density of the fluid in the cochlea were set to 2250 MPa and 1000 kg/m^3^, respectively. The material properties used for the cochlea were summarized in Table 3.

### 2.3. Boundary Conditions

The end nodes of all the muscles and ligaments were fixed to zero displacement, including the periphery of the stapedial annulus ligament. The outer edge of tympanic membrane annulus was defined as the fixed constraint. The surfaces of the elements that represent the bony wall of the cochlea were set as rigid walls. A uniform pressure was applied on the eardrum to simulate the sound stimulus of normal human ear.

### 2.4. FE Modeling of the RW Stimulation

As our main concern is the influence of the otosclerosis on the RW stimulation's performance, not the effects of the MEI's parameters, the actual structure of the MEI's actuator was not modeled in our model for simplification. The RW stimulation was simulated by applying driving force onto the surface of RW along the normal direction of the RW.

In the design of the middle ear implant, it is important to quantify its hearing compensating performance. The stapes displacement is usually used as the reference parameter in this evaluating process. However, the stapes lies outside the cochlea. Its vibration cannot reflect the inner dynamical properties of the cochlea accurately. As the intracochlear differential pressure across the cochlear partition at the base of the cochlea is proved to be a sensitive measure of the cochlear input [29] and the vibration of the basilar membrane can be deduced by the intracochlear pressure [30, 31], the intracochlear pressure was measured by many researchers to uncover the mechanism of cochlear mechanics [29, 32, 33] and evaluate the middle ear implant's performance [13, 34, 35]. Thus, in this paper, we also used the intracochlear pressure to assess the round window stimulation's performance. Specifically, we used the equivalent sound pressure (ESP), which is expressed as [13]

(1) $$\begin{array}{c} P_{eq}=90+20\log\left(\;\frac{\left|P_{SV}^{R}-P_{ST}^{R}\right|}{\left|P_{SV}^{F}-P_{ST}^{F}\right|}\;\right), \end{array}$$

where *P*_SV_^*F*^, *P*_ST_^*F*^ are the fluid pressures in the cochlear scala vestibule and scala tympani under forward sound stimulation (90 dB SPL applied onto the eardrum) and *P*_SV_^*R*^, *P*_ST_^*R*^ are the fluid pressures in the scala vestibule and scala tympani under reverse RW stimulation (50 *μ*N driving force applied onto the round window membrane [12, 13]). The detailed derivation of (1) can be found in [13].

### 2.5. Simulation of Otosclerosis in FE Model

Otosclerosis is a disease with abnormal growth of the ossicular bones in the middle ear. The typical changes of otosclerosis in the human ear are increased stiffness of the stapedial annular ligament, stapedial abnormal bone growth, and partial fixation of the malleus. To simulate the enlargement of the stapedial annular ligament's stiffness and the partial fixation of the malleus, the elastic modulus of the normal stapedial annular ligament (SAL) and the anterior mallear ligament (AML) were increased by a factor of 10 and 100, respectively. In terms of the stapedial abnormal bone growth, we boosted the FE model's stapes mass by a factor of 5 to mimic this symptom, based on the literature [19].

## 3. Results

### 3.1. Convergence Test

**Figure 2:**
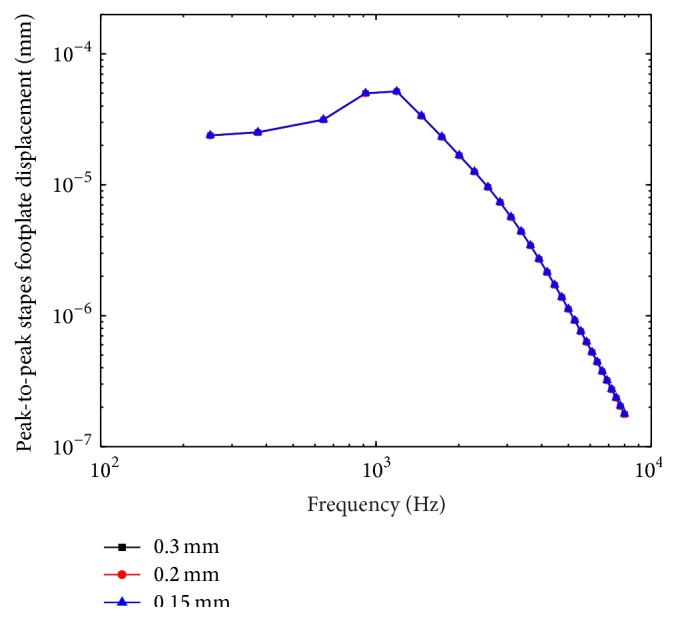
Convergence test for the human ear FE model.

Convergence test is used to investigate what mesh resolution should be used in our FE models. We established a set of 3 models in which the uniform sizes of the elements are 0.3 mm, 0.2 mm, and 0.15 mm, respectively. These models were compared based on the model-predicted stapes displacement under 90 dB sound pressure applied at the eardrum. Result of this test is plotted in Figure 2. It shows that these three models have almost the same stapes displacement curves. The maximum difference between the 0.2-mm model and the 0.15-mm model was less than 0.7%. This result implied that the 0.2-mm model provides enough accuracy and it is the one used for the remaining simulations.

### 3.2. Human Ear FE Model Verification

**Figure 3:**
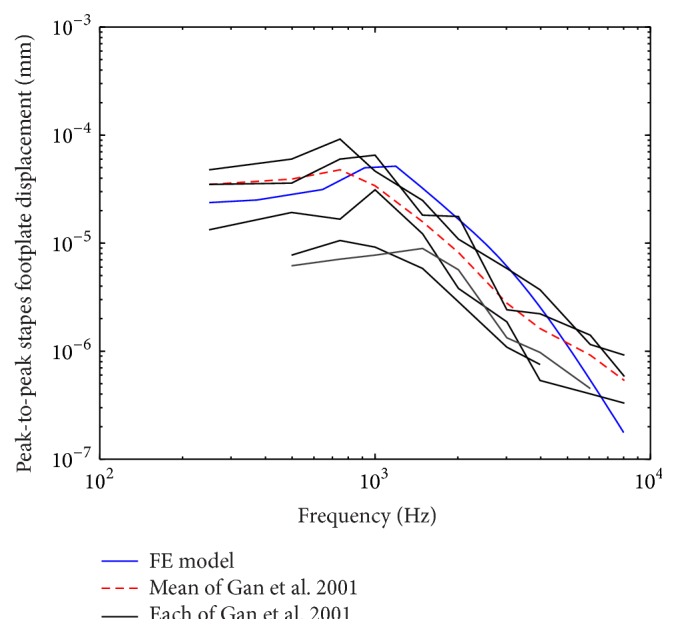
Comparison of the FE model-predicted stapes footplate displacement with the experimental data.

**Figure 4:**
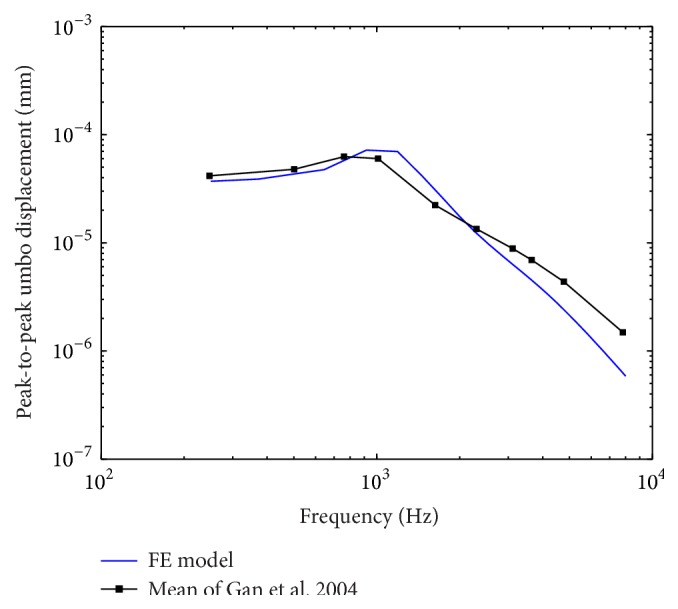
Comparison of the FE model-predicted umbo displacement with the experimental data.

In order to establish the extent to which the prediction of the human ear FE model accords with reality, comparisons against four sets of experimental studies were done. Gan et al. experimental data of stapes footplate displacement [36] and umbo displacement [37] obtained from temporal bones were initially selected for the model verification. A uniform pressure of 90 dB SPL on the lateral side of the eardrum was applied to our human ear FE model. A harmonic analysis was conducted on the model. As the most important frequencies for speech fall into the 250–6000 Hz range, and the range between 250 Hz and 8000 Hz is normally used in the audiogram, the harmonic analysis focused on the range between 200 Hz and 8000 Hz. The model-predicted results were plotted with the experimental curves in Figures 3 and 4.  Figure 3 shows that our model-predicted stapes displacement curve lies close to the mean of the experimental curves. Likewise, the FE model-predicted umbo displacement is close to the mean experimental curve as shown in Figure 4. But, at high frequency, both the model-predicted displacements of the stapes and the umbo are small compared to their corresponding mean experimental curves. This discrepancy may be attributed to the constant elastic modulus and Rayleigh type damping we used, which makes the damping of the soft tissues in our model proportional to the frequency as well as too high at the high frequency [14].

**Figure 5:**
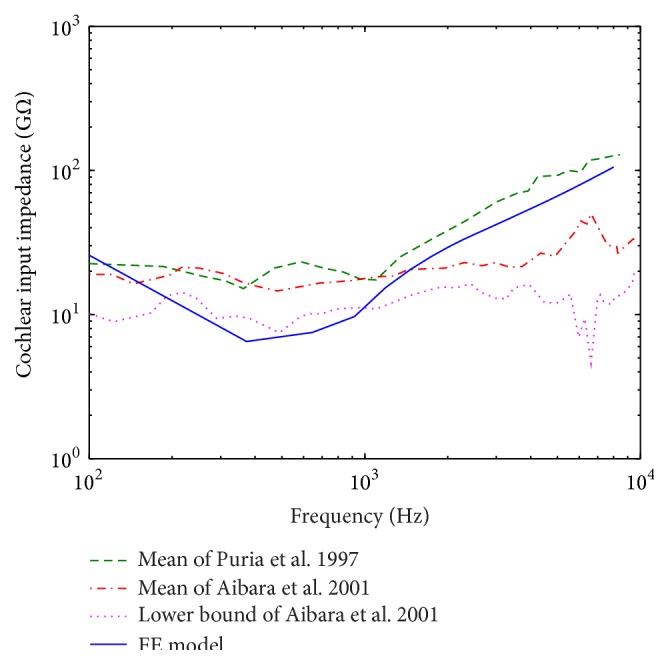
Comparison of the FE model-predicted cochlear input impedance with that measured on human temporal bones by Puria et al. [38] and Aibara et al [39].

The cochlear input impedance, defined as the ratio of sound pressure produced in the scala vestibuli at the oval window to the volume velocity of scalae fluid at the oval window, is an important parameter reflecting the sound energy transfer property from the middle ear into the cochlea. Thus, the experimental data of the cochlea input impedance published by Puria et al. [38] and Aibara et al. [39] were also selected for current model evaluation. The model-predicted cochlear input impedance was plotted with the experimental curves in Figure 5. As shown in Figure 5, the model-predicted results are in reasonable agreement with the published data in terms of overall response trends. The model-derived value deceased from 100 Hz to 380 Hz with a minimum of 6.5 GΩ at 380 Hz. However, at frequencies above 380 Hz, the model-derived impedance increases gradually with the frequency. In general, the FE results are more consistent with the impedance data obtained by Puria et al., especially at frequencies higher than 1 kHz.

**Figure 6:**
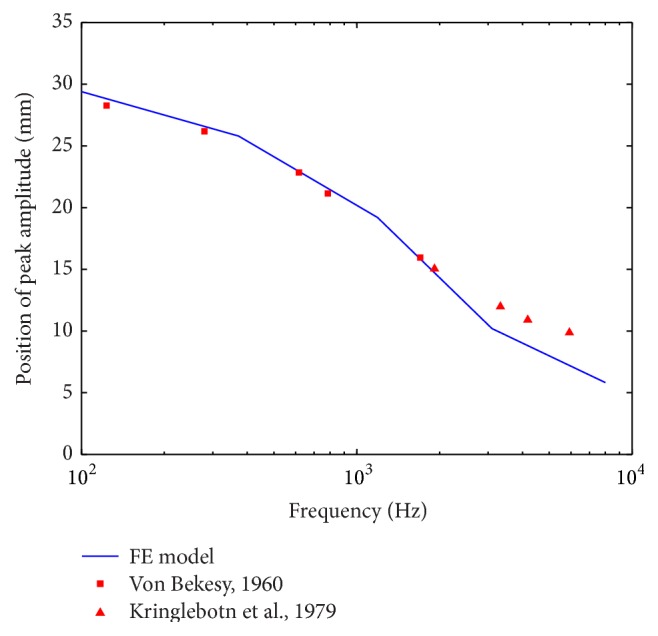
Comparison of the FE model-predicted cochlear best frequency map with experimental reports.

Cochlear best frequency (BF) map indicates the frequency corresponding to the peak BM vibration as a function of location along the BM length. This BF map demonstrates the cochlear frequency selectivity.  Figure 6 shows our model-predicted BF map compared with the experimental curves reported by Von Bekesy [40] and Kringlebotn et al. [41]. As shown in Figure 6, a reasonable agreement exists between our model-predicted results and these measurements.

These above comparisons show that our human ear FE model's predictions, in general, match experimental results obtained from human temporal bones. Therefore, this FE model is able to predict biomechanical characteristics of the human ear system.

### 3.3. Effect of the SAL Stiffness Enlargement on the RW Stimulation Performance

**Figure 7:**
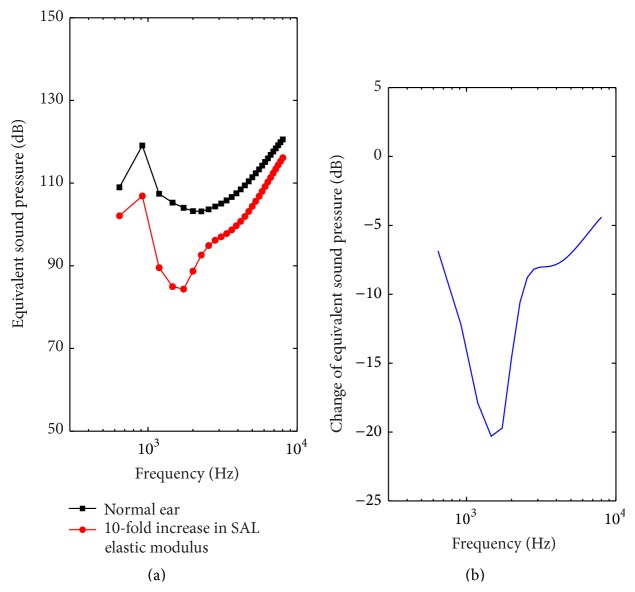
The effect of the SAL stiffness enlargement on the RW stimulation performance. (a) Equivalent sound pressure (ESP). (b) Change of the equivalent sound pressure with the augment of the SAL elastic modulus.

The effect of the SAL stiffness enlargement on the round window stimulation performance was investigated by comparing the ESP corresponding to an excitation force of 50 *μ*N on the round window membrane under different SAL elastic modulus. To study the effect of the stiffness enhancement related to the otosclerosis, we increased the elastic modulus of the SAL by 10-fold [42].  Figure 7(a) shows that the increase of the SAL stiffness decreases the ESP of the RW stimulation over the entire frequencies. Besides, this decrease is significant at lower frequencies (below 3 kHz). The maximum decline of the ESP is about 20 dB at 1400 Hz (Figure 7(b)). At higher frequencies (>3 kHz), the adverse effect of the SAL stiffness enlargement on the ESP decreased with the increase of the frequency.

### 3.4. Effect of the Stapedial Abnormal Bone Growth on the RW Stimulation Performance

**Figure 8:**
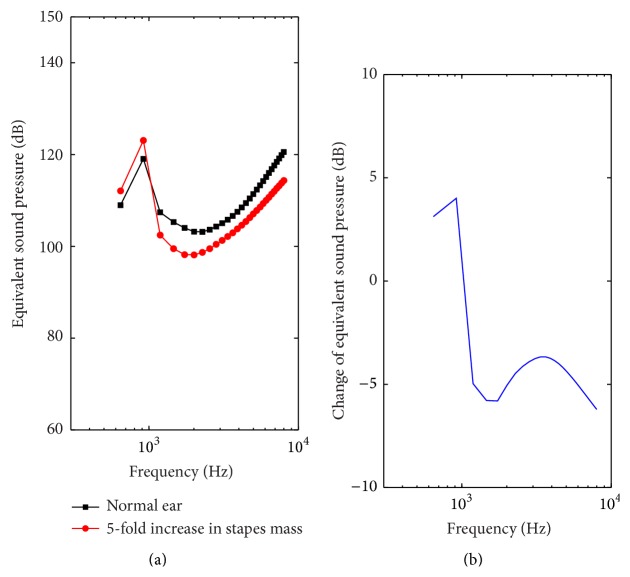
The effect of the stapedial mass augment on the RW stimulation performance. (a) Equivalent sound pressure (ESP). (b) Change of the equivalent sound pressure with the augment of the stapedial mass.

The stapedial abnormal bone growth was simulated by increasing the mass of the stapes [19]. And the influence of stapes mass change on the round window stimulation performance was studied (Figure 8). By increasing the density of stapes from 2.2 × 10^3^ kg/m^3^ to 1.1 × 10^4^ kg/m^3^, we got the simulated mass of the stapes boosted by 5-fold. The model-predicted result (Figure 8(a)) shows that the increase of the stapes mass has little impact on the ESP at lower frequencies (below 900 Hz). However, when the frequency increases, the ESP starts to decrease with the addition of the stapes mass. The maximum drop in ESP is around 6.2 dB at 8000 Hz (Figure 8(b)).

### 3.5. Effect of the Partial Fixation of the Malleus on the RW Simulation Performance

**Figure 9:**
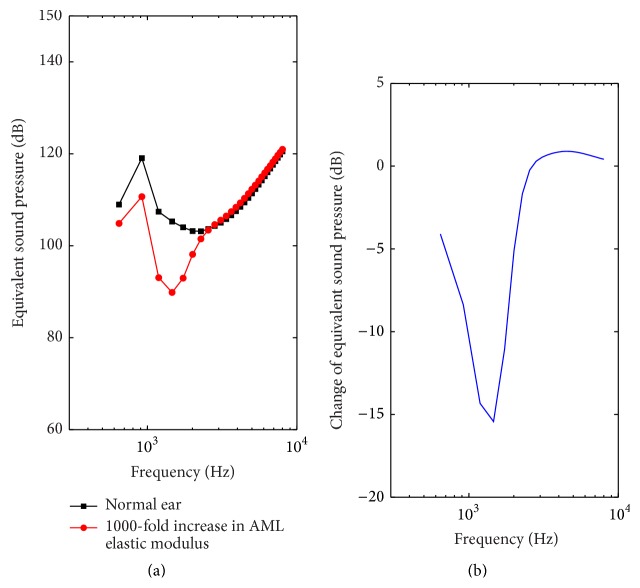
The effect of partial fixation of the malleus on the RW stimulation performance. (a) Equivalent sound pressure (ESP). (b) Change of the equivalent sound pressure with the 1000-fold increase in AML elastic modulus.

The effect of the partial fixation of the malleus on the RW stimulation performance was investigated by change of the elastic modulus of the AML [16]. To simulate partial mallear fixation with a calcified AML, the elastic modulus of normal AML (2.1 × 10^7^ MPa) was increased by a factor of 1000 [16].  Figure 9 shows the corresponding model-predicted results. Similar to SAL stiffness's effect, the increase of the AML elastic modulus does not impact the ESP significantly at higher frequencies above 3000 Hz (Figure 9(a)). However, the RW stimulation ESP with AML enlargement showed a significant drop in the lower frequencies (below 3000 Hz), especially in the range of 1000 Hz to 2000 Hz. The maximum drop in ESP is about 15.4 dB at 1500 Hz, where a notch is present (Figure 9(b)).

## 4. Discussion

Coupling the MEI's actuator directly to the cochlear round window, which bypasses the ossicular chain, is a new application of middle ear implant for the treatment of patients with middle ear disease. Clinical reports show a considerable variability (30 dB) in patients' outcomes. Potential variables, which have been identified as the reason for this outcomes, can be classified into device-loading and patient-specific variables. The main device-loading variables include the different orientation of the IMEHD's actuator, the intervening materials between the RW and the actuator, the overlaying materials, and the loading pressure applied to the RW. Lupo et al.'s experimental report [43] demonstrates that the loading pressure and angle of approach do not have a significant effect on the MEI's performance. Based on temporal bone experiment, Arnold et al.'s study [44] indicates that underlying a connective tissue between the RW and the actuator can get a substantial improvement of up to 13 dB. Besides, comparing with the effect of the intervening materials, the influence of the overlaying materials is considerably smaller. All of above researches only focus on the device-loading variables.

Considering that the treatment of otosclerosis is one of the main targets of the RW stimulation, the patient-specific variables corresponding to the otosclerosis were also investigated in this paper. Three typical changes of ear components corresponding to otosclerosis in the auditory system were studied in our FE model: stapedial annular ligament stiffness enlargement, stapedial abnormal bone growth, and partial fixation of the malleus.

Based on experiments and middle ear mechanical models, Huber et al. [16] and Feng and Gan [42] both found that the SAL stiffness enlargement deteriorates normal hearing perception dramatically at lower frequencies. In terms of its influence on RW stimulation, our results show the same trend; that is, the SAL stiffness augment mainly deceased RW simulation performance at lower frequencies below 3000 Hz (about 20 dB at 1400 Hz). The enlargement of SAL stiffness shows a similar influence on normal hearing perception and RW stimulation performance may be attributed to the fact that the stiffness impedes the motion at low frequencies in dynamic system. Besides, the 20 dB deterioration is considerably large regarding the clinical reported variability in RW stimulation's outcomes (30 dB). Therefore, to cure this kind of disease, the influence of the SAL stiffness enlargement cannot be ignored, and the corresponding MEI's driving force should be strengthened prominently at lower frequencies in the design of this type of MEI.

With regard to the stapedial abnormal bone growth, we increased the mass of stapes by 5 times to simulate this symptom [19]. Our results show that the augment of stapedial mass increased the RW stimulation performance slightly at lower frequencies below 1000 Hz, while decreasing the performance severely at higher frequencies above 1200 Hz. This is mainly because the adding mass of the stapes shifts the resonance of the middle ear system toward lower frequencies. The maximum drop in ESP, which is around 6.2 dB at 8000 Hz, is relatively small compared with the influence of the enlargement of SAL stiffness (20 dB). To simulate the symptom of partial mallear fixation, Huber et al. increased the AML's elastic modulus by a factor of 1000 [16]. In this paper, we utilized the same method to model this disease. Similar to the influence of the SAL stiffness enlargement, the partial mallear fixation also deteriorates RW stimulation performance prominently at lower frequencies below 3000 Hz. The maximum drop in ESP is about 15.4 dB. This deterioration level is much larger than the stapedial mass's influence but comparable to the effect of the SAL stiffness enlargement.

The above results demonstrate that the typical characteristics of the otosclerosis do decrease the RW stimulation performance, particularly at lower frequencies below 3000 Hz. Considering that we perceive the speech mainly in the frequency range of 500 Hz to 3000 Hz [45, 46], this deterioration on lower frequency region (below 3000 Hz) is detrimental to the patients' speech understanding. Thus, the driving capacity of the RW stimulation should be enhanced, particularly at lower frequencies below 3000 Hz.

## 5. Conclusion

In this study, a FE model of the human ear including the middle ear and cochlea was used to investigate the influence of otosclerosis on RW stimulation. The FE model was constructed based on a complete set of computerized tomography section images of a right ear by reverse engineering technology, and its validity was verified by comparing the model-predicted results with published experimental data. The results show that the enlargements of stapedial annular ligament and partial fixation of the malleus decrease RW stimulation's ESP prominently at lower frequencies. In contrast, the stapedial abnormal bone growth deteriorates RW stimulation's ESP severely at higher frequencies.

## Figures and Tables

**Table 1:** Dimensions of the human ear finite element model.

Structure	Our model	Published data
*Eardrum*		
Diameter along manubrium	8.99 mm	8.0–10.0 mm [47]
Diameter perpendicular to manubrium	7.75 mm	7.5–9.0 mm [47]
Thickness	0.1 mm	0.1 mm [47]
*Malleus*		
Total length	7.67 mm	7.6–9.1 mm [47]
*Incus*		
Length along long process	7.10 mm	7.0 mm [47]
Length along short process	4.89 mm	5.0 mm [47]
*Stapes*		
Height	3.75 mm	2.5–4.0 mm [47]
Length of footplate	2.95 mm	2.64–3.36 mm [47]
Width of footplate	1.51 mm	0.7–1.66 mm [47]
*Cochlea*		
Length of cochlea	37 mm	29.07–37.45 mm [48]
Length of BM	34 mm	35 mm [49]
Width of BM at base	0.15 mm	0.1 mm [49]
Width of BM at apex	0.5 mm	0.5 mm [49]
Thickness of BM at base	7.5 *μ*m	7.5 *μ*m [50]
Thickness of BM at apex	2.5 *μ*m	2.5 *μ*m [50]
Volume of scala vestibuli	92.315 mm^3^	90.8463 mm^3^ [51]
Volume of scala tympani	93.270 mm^3^	91.2044 mm^3^ [51]
Area of RWM	2.1 mm^2^	2.08 mm^2^ [21]

**Table 2:** Material properties of the middle ear components.

Structure	Young's modulus (N/m^2^)	Density (kg/m^3^)
*Eardrum*		
Pars flaccida	1.00 × 10^7^	1.20 × 10^3^
Pars tensa	3.20 × 10^7^	1.20 × 10^3^
*Ossicles*		
Malleus		
Head	1.41 × 10^10^	2.55 × 10^3^
Neck	1.41 × 10^10^	4.53 × 10^3^
Handle	1.41 × 10^10^	3.70 × 10^3^
Incus		
Body	1.41 × 10^10^	2.36 × 10^3^
Short process	1.41 × 10^10^	2.26 × 10^3^
Long process	1.41 × 10^10^	5.08 × 10^3^
Stapes	1.41 × 10^10^	2.20 × 10^3^
*Joint*		
Incudomalleolar joint	1.41 × 10^10^	2.39 × 10^3^
Incudostapedial joint	4.00 × 10^6^	1.20 × 10^3^
*Ligament/tendon*		
Tympanic annulus ligament	4.00 × 10^5^	1.20 × 10^3^
Superior mallear ligament	4.90 × 10^6^	1.20 × 10^3^
Lateral mallear ligament	6.70 × 10^6^	1.20 × 10^3^
Anterior mallear ligament	2.10 × 10^7^	1.20 × 10^3^
Superior incudal ligament	4.90 × 10^6^	1.20 × 10^3^
Posterior incudal ligament	6.50 × 10^6^	1.20 × 10^3^
Stapedial annulus ligament	4.10 × 10^5^	1.20 × 10^3^
Tensor tympani tendon	2.60 × 10^6^	1.20 × 10^3^
Stapedial tendon	5.20 × 10^5^	1.20 × 10^3^

**Table 3:** Material properties of the cochlear components.

Structure	Our model	Published data
*Round window membrane*		
Density (kg/m^3^)	1.20 × 10^3^	1.20 × 10^3^ [22]
Young's modulus (MPa)	0.3	0.3 [13], 0.35 [22]
*Basilar membrane (BM)*		
Density (kg/m^3^)	1.20 × 10^3^	1.20 × 10^3^ [22]
Young's modulus (MPa)		
At the base	40	40 [13], 50 [22]
In the middle	15	15 [22]
At the apex	3	3 [22]
*Perilymph fluid*		
Density (kg/m^3^)	1.00 × 10^3^	1.00 × 10^3^ [22]
Bulk modulus (MPa)	2.25 × 10^3^	2.20 × 10^3^ [22]
*Osseous spiral lamina*		
Density (kg/m^3^)	1.20 × 10^3^	1.20 × 10^3^ [22]
Young's modulus (MPa)	1.41 × 10^4^	1.41 × 10^4^ [22]
